# Magnetic Nano-Sized SDF-1 Particles Show Promise for Application in Stem Cell-Based Repair of Damaged Tissues

**DOI:** 10.3389/fbioe.2022.831256

**Published:** 2022-04-27

**Authors:** Xu Wang, XinXin Han, Yi Qiu, Jianbo Sun

**Affiliations:** ^1^ The First Dongguan Affiliated Hospital, Guangdong Medical University, Dongguan, China; ^2^ Bioland Laboratory (Guangzhou Regenerative Medicine and Health Guangdong Laboratory), Guangzhou, China; ^3^ The Fifth Affiliated Hospital, Guangzhou Medical University, Guangzhou, China; ^4^ Hospital of Stomatology, Guanghua School of Stomatology, Sun Yat-Sen University, Guangzhou, China; ^5^ Guangdong Provincial Key Laboratory of Stomatology, Sun Yat-sen University, Guangzhou, China; ^6^ Zhongshan School of Medicine, Sun Yat-Sen University, Guangzhou, China

**Keywords:** stromal cell-derived factor-1 SDF-1, stem cell homing, magnetic nanoparticle MNP, tissue repair, bone regeneration

## Abstract

Stem cell-based therapy is a promising option for repair of injured tissue. Stem cells have homing characteristics and can be mobilized to the injury sites following activation, under the regulation of the SDF-1/CXCR4 axis. However, a sufficient level of stem cell aggregation and retention is essential for ensuring favorable repair outcomes. Problems related to stem cell delivery/recruitment efficiency and retention in the injury site are among the main challenges faced during *in vivo* studies on stem cell therapy. In this study, we designed an SDF-1(alpha) magnetic nanoparticle delivery system for stem cell recruitment. We expressed and purified a biotin-labeled SDF-1(alpha) protein and immobilized it on streptavidin-modified magnetic nanoparticles (MNP) through the streptavidin–biotin linkage, with an efficiency of approximately 14%. The physicochemical properties of the SDF-MNP in glycerol buffer were similar to those of the streptavidin-modified MNP. Further evidence suggested that SDF-MNP barely show cytotoxicity even at a concentration of 125 µg/ml MNP and have a promising chemotaxis effect on mesenchymal stem cells *in vitro* and *in vivo*. Our study provides a strategy for the assembly of magnetic nanoparticle carrier systems for protein factors, as well as preliminary evidence for the application of SDF-MNP in stem cell-based therapy for the regeneration of injured bone tissue.

## Introduction

Stem cells have the ability to continuously renew,replicate, and differentiate into tissue cells under appropriate conditions or microenvironments. Mesenchymal stem cells (MSCs) are among the stem cell populations displaying low immunogenicity, and some kind of MSCs in circumstance can be amplified to numbers (Kangari et al., 2020). These properties make MSCs ideal for use in tissue regeneration and repair of the tooth and cranial bone, among others (Singer and Caplan, 2011). Repair of the bone or tooth damage has mainly been carried out using expensive synthetic inorganic materials. In contrast, the direct application of MSCs at the target site presents as a more cost-effective and natural way of repairing the tissue, without the use of synthetic materials (Gkouma et al., 2021).

Stem cells exhibit important biological functions, mainly after they reach a sufficient number. Therefore, aggregating stem cells at the target site to induce their biological function is one the biggest challenges in the application of stem cell therapy for the repair of tissue damage (Ho-Shui-Ling et al., 2018). Various strategies exist for the enrichment of stem cells at the injury site. In one such strategy, the researchers transplanted stem cells labeled with magnetic nanoparticles and then applied a magnetic field to the injured site to increase the concentration of the stem cells at the target site for an improved therapeutic efficacy (Yun et al., 2018). Another way of increasing stem cell number at the target site involves attaching the stem cells to scaffold materials prior to transplantation to the injury site (Long et al., 2014). Alternatively, the naturally induced activation and mobilization of stem cells is also considered desirable to facilitate a continuous supply of stem cells for tissue regeneration.

MSCs have homing abilities, which facilitate their migration to the damage sites (Krueger et al., 2018). The processes and mechanisms of stem cell homing have attracted much attention. MSC homing consists of three processes: rolling/tethering, stable adhesion, and migration (Wise and Ricardo, 2012). Another non-negligible mechanism is MSCs are often to be retained by simple entrapment (Kavanagh et al., 2015). Multiple cytokines are involved in the homing process. Among them, the human stromal cell-derived factor-1 (SDF-1(alpha)) and its receptor C-X-C motif chemokine receptor 4 (CXCR4) are considered to form the key signaling axis affecting the homing of stem cells (Marquez-Curtis and Janowska-Wieczorek, 2013). SDF-1(alpha) is also alternatively known as C-X-C motif chemokine ligand 12 (CXCL12). The expression of the gene encoding SDF-1(alpha) among those encoding the alpha-chemokine family of proteins is observed in various cells (Nagasawa, 2014). CXCR4 is a G-protein-coupled transmembrane receptor of SDF-1(alpha) and is expressed in various cells (Janssens et al., 2018). MSCs highly express CXCR4; the binding of SDF-1(alpha) to CXCR4 activates extracellular signal-regulated kinases and the phosphorylation of phosphatidylinositol 3-kinase (PI3K), initiating multiple signaling pathways to induce cell migration (Roland et al., 2003). However, it has been observed that typically, a low number of transplanted or endogenous MSCS migrate to the target site (Marquez-Curtis and Janowska-Wieczorek, 2013).

Given the key role of the SDF-1(alpha)/CXCR4 signaling axis in stem cell nesting, the number of homing MSCs has been observed to increase following genetic modifications that either increased SDF-1(alpha) levels *in situ* or enhanced the CXCR4 expression of stem cells, or following the implantation of a scaffold with immobilized SDF-1(alpha) (Roland et al., 2003; Shen et al., 2016). However, the gene manipulation techniques pose the risk of inadvertent genetic modification or the tumor transformation of a large number of stem cells. In addition, the SDF-1(alpha) injected at the target site may dissipate systemically, leading to insufficient concentrations at the target site and the loss of the expected effect.

Recently, treatment of rats with SDF-1(alpha) immobilized (through use of heparin) on non-toxic genipin (Gp) cross-linked chitosan (CHI) scaffolds showed better wound recovery rates along with higher vascular endothelial growth factor (VEGF) and transforming growth factor beta (TGF-β) expression in the surrounding tissue of the injury site (Yao et al., 2020). Application of SDF-1(alpha) by loading it in chitosan (CS)/β-glycerophosphate disodium salt pentahydrate (βGP) hydrogels containing appropriate concentrations of hyaluronic acid (HA) showed a sustained release of SDF-1(alpha) and enhanced adipose stem cell migration and retention (Fadera et al., 2020). Similarly, the use of fabricated SDF-1(alpha)–gelatin/hyaluronate (Gel/HA) copolymer–hydroxyapatite (HAP) scaffolds for tissue repair in a rat femoral condyle bone defect model showed faster bone growth compared to the observations in the composite without the SDF-1(alpha) group (Chang et al., 2021). These gel-based packages with SDF-1(alpha) showed no obvious toxicity or side effects after material implantation (Fadera et al., 2020; Chang et al., 2021).

Use of target-controlled nanomaterials for carrying SDF-1(alpha) to the target site to directly induce stem cell migration and abundance is an effective strategy to solve the bottleneck of stem cell therapy. In this study, we constructed a nanoscale magnetic SDF-1(alpha) particle using a streptavidin-biotin affinity system. Preliminary results regarding its effects on MSC migration and retention suggest that this magnetic SDF-1(alpha) nanoparticle system has potential application prospects in stem cell therapy for tissue repair.

## Materials and Methods

### Ethics Statement

This study was approved by the Animal Ethics Committee of Sun Yat-sen University. All mice experiments were conducted in strict accordance with the Guidelines for the Care and Use of Laboratory Animals proposed by the National Institutes of Health.

### Experimental Animals

Male BALB/c mice (8–10 weeks old), purchased from the Experimental Animal Center of the East Campus of Sun Yat-sen University, were used to create the bone fracture model. The mice were housed in a specific pathogen-free environment.

### Plasmid, Bacterial Strain, and Mammalian Cells

The primers, plasmids, and bacterial strains used in this study are listed in Supplementary Table S1. The pUC19 T simple vector was used to construct the amplified plasmid containing the target genes. The pET28a(+) and pCDFDuet-1 plasmids were used to construct a mouse SDF-1(alpha) isoform expression plasmid. *SDF* (primer with a *Bap* DNA sequence) and *BirA* were amplified from the mouse bone marrow cell cDNA and *Escherichia coli* DH5α genomic DNA, respectively. The *SDF-1* gene was inserted into multiple cloning sites of pET28a(+). *BapSDF* and *BirA* were inserted into multiple cloning sites of the pCDFDuet-1 plasmid. The expression plasmid was transformed into *E. coli* BL21 (DE3) cells. *E. coli* was cultured in Luria broth (LB) at 37°C with shaking at 200 rpm. For protein induction, the activated bacterial strain was inoculated in LB. After the medium reached an OD_600_ of 0.6∼0.8, IPTG (final concentration 0.5 mM or 1 mM) induction was carried out at 30°C for 4 h. Antibiotics were used at the following concentrations (µg/ml): ampicillin (Amp), 100; kanamycin (Km), 50; and streptomycin (Sm), 50. MSCs (MSCs used in this study were induced with pluripotent stem cell (iPSC)-MSCs (Chang et al., 2019), given by Fu Qingling’s laboratory in The First Affiliated Hospital of Sun Yat-sen University) were cultured in Dulbecco’s modified Eagle’s medium containing 10% FBS, 1 μg/L bFGF, 10 μg/L EGF, L-glutamax, NEAA, 55 μM beta-mercatoethanol, and penicillin/streptomycin (Sun et al., 2012).

### Purification of His-Tag Proteins

The recombinant *E. coli* bacterial strain was used to express the target protein. The cells were collected by centrifugation. A 1/10 volume of lysis buffer containing 1×PBS (pH 7.4 unless specifical mention), 0.1% Triton X-100, and 20 mM imidazole was added, and the pellet was resuspended. The cells were then lysed by ultrasonication. The supernatant was collected after the removal of the cell debris. This solution was incubated with nickel agarose gently to ensure better binding of the two components. The nickel agarose gel was then treated with an adequate amount of wash buffer containing 1 ×PBS (pH 7.4) and 40 mM imidazole to remove non-specifically bound proteins. The target proteins were eluted with an elution buffer containing 1×PBS (pH 7.4) and 500 mM imidazole. The target proteins were detected by SDS-PAGE. For carrying out the denaturing purification of the proteins, 8 M urea was added to the lysis buffer, wash buffer, and elution buffer. The eluted protein solution was dialyzed with a glycerol-TEN (GTEN) dialysis buffer containing 50 mM Tris (pH 7.9), 0.5 mM EDTA, 50 mM NaCl, and 5% glycerol with a decreasing gradient of imidazole and urea to refold the denatured protein. Purified protein was added into a dialysis bag and then dialyzed in 4 M urea GTEN buffer at 4°C for 1.5 h. Then, it was replaced by 2 M urea buffer by adding 1 volume non-urea GTEN buffer and incubated at 4°C for 1.5 h. Then, the buffer was replaced by adding 1 volume GTEN buffer incubated at 4°C for 1.5 h until the concentration of urea decreased to less than 0.1 M. The non-urea GTEN buffer was added and incubated at 4°C overnight. Then, the protein was collected and centrifuged. The supernatant contained refolded protein and can be used in experiment or stored at −20°C.

### Western Blotting and Enzyme-Linked Immunosorbent Assay

For western blotting, SDS loading buffer was added and incubated at 95°C for 10 min. The proteins were separated by SDS-PAGE and transferred to a polyvinylidene difluoride (PVDF) film, followed by blocking with 5% non-fat milk (or BSA). The primary antibodies and the HRP-conjugated secondary antibody were incubated with the PVDF film. The target protein on the PVDF film was visualized using an ECL agent and a chemical illumination imaging system. For the ELISA experiment, the proteins were coated in the wells with overnight incubation in the coating buffer. Next, the wells were blocked with 1% BSA PBS buffer and washed with PBS containing 0.05% v/v Tween 20. The primary antibodies and the HRP-conjugated secondary antibody were incubated, followed by the addition of the TMB substrate. Then, 0.2 M sulphuric acid was added, and absorbance at 450 nm was read using a microplate spectrophotometer. The antibodies used in this study included mouse anti-His antibody (TIANGEN Biotech) for the detection of His tag and rabbit anti-SDF-1(alpha) antibody (Abcam) for the detection of recombinant mouse SDF protein. For western blotting and ELISA of biotinylated proteins, HRP-conjugated Sa (Solarbio) was used. For the ELISA of SDF-MNP, the particle was directly coated in the microtiter wells, and the remainder of the procedure was carried out as per the standard ELISA protocol.

### Conjugation of Sa-MNP and BapSDF (Biotin)

1 ml 1 mg/ml Sa-MNP suspension (30 nm, Ocean NanoTech) was collected using magnetism (herein termed as magnetic separation). The Sa-MNP were resuspended in 1 ml BapSDF (biotin) solution by ultrasonication. The reaction system was then stirred at 200 rpm and 30°C for 1 h. Thereafter, SDF-MNP were magnetically separated and washed twice with 1 ml PBS or GTEN buffer. The SDF-MNP were finally resuspended in PBS or GTEN buffer for storage at 4°C.

### Particle Diameter Measurements

MNP (Sa-MNP or SDF-MNP) were magnetically separated and resuspended in buffer at a concentration of 1 µg/ml (all buffers were first passed through a 0.22 μm filter). The MNP particles were fully separated from each other by ultrasonication. At least, 500 µl of the MNP suspension was used. Samples were detected using a Malvern Panalytical NanoSight NS300 (Malvern), and data were analyzed using Nanoparticle Tracking Analysis software.

### Perls Staining

iPSC-MSCs were passaged and cultured overnight to a confluence of approximately 60%. Equal volumes of MNP suspension of different concentrations were added. After culturing for 24 h, the cells were washed twice with PBS and then fixed with 4% PFA for 10 min, followed by washing with PBS at room temperature. The cells were then stained according to the manufacturer’s instructions. After drying in air, the plates were observed and imaged using a microscope.

### Cytotoxicity Detection

iPSC-MSCs were cultured to reach the logarithmic phase and passaged at a density of 2–5 × 10^4^/ml into a 96-well plate. After culturing for 2–4 h, SDF-1(alpha) or MNP was added followed by a subsequent 24 h cultivation. The supernatant was aspirated and a new complete medium containing a 10% CCK-8 agent was added. After 1–4 h of culture, the OD of the medium was measured at 450 nm using a microplate spectrophotometer. To eliminate reducibility of MNP which may have an effect on the signal of the CCK-8 test, an MNP/FBS medium without cell was set as control. The final detectable value of CCK-8 was calculated by the OD_450_ value of the experimental test (containing MNP and cell) minus the value of the control test (containing only MNP).

### Transwell Experiment

iPSC-MSCs were cultivated to a confluence of approximately 80%–90%. Cells were digested and separated using 0.05% Trypsin-0.02% EDTA solution. Cell density was measured and adjusted to 1 × 10^5^/ml. The cell suspension (200 µl) and medium (700 µl) containing SDF-1(alpha) or MNP were added to the transwell chamber and the 24-well plate, respectively. Then, the cells were cultured for 24 h, and the suspension in the transwell was discarded. The transwell was then washed with PBS. Then, the cell debris on the transwell membrane was completely removed using a cotton swab. Cells on the lower side of the membrane were fixed and stained with 0.1% crystal violet. The cells were detected and imaged under a microscope.

### Femoral Injury Model and *In Vivo* Imaging of the Mice

MSCs were cultivated and labeled by the Vivo Track 680 near-infrared (NIR) Fluorescent Imaging Agent (PerKinElmer) according to the manufacturer’s instructions. The labeled cells were resuspended, and the cell density was adjusted to 1 × 10^7^ cells/ml. The cells were transplanted into mice by tail injection at a concentration of 2 × 10^6^ cells/200 μl per mouse. After a recovery period of 1 h, the mice were anesthetized by inhalation of ether, and femoral injury was induced by a fracture in the middle region of the femur. For the femoral injury model *in vivo* test, the thin steel was crossed through a syringe needle; then, the femur marrow cavity was stabbed with them. The needle was pulled out and the thin steel was left in. It gently fractured the femur in middle, and the thin steel can help keep the two broken bones together instead of total separation which is good for bone rebuilding. SDF-MNP or the control buffer were injected into the injury site. Hollow ring magnetism was applied at the injection site for half an hour. The mice were imaged, and the signal was analyzed using small animal *in vivo* imaging (SAIVI) after 1, 3, and 9 days of feeding.

### Statistical Analyses

Data were presented as mean ± standard deviation. Statistical significance was determined by two-tailed Student’s t-test by GraphPad prism 8. *p* value < 0.05 was considered statistically significant. *p* > 0.05: ns, *p* < 0.05: *, *p* < 0.01: **, *p* < 0.005: ***, and *p* < 0.0001: ****.

## Results

### Expression of Biotinylated SDF (BiotinSDF)

To assemble a magnetic nanoscale SDF-1 (alpha) utilizing biotin–Sa linkage, we used biotin-modified SDF and Sa-modified MNP (streptavidin-modified magnetic nanoparticle) to achieve our purpose. As the Bap peptide can be modified with biotin using BirA, we first constructed a BapSDF (co-expression with BirA)-expressing strain and purified this recombinant protein.

**FIGURE 1:**
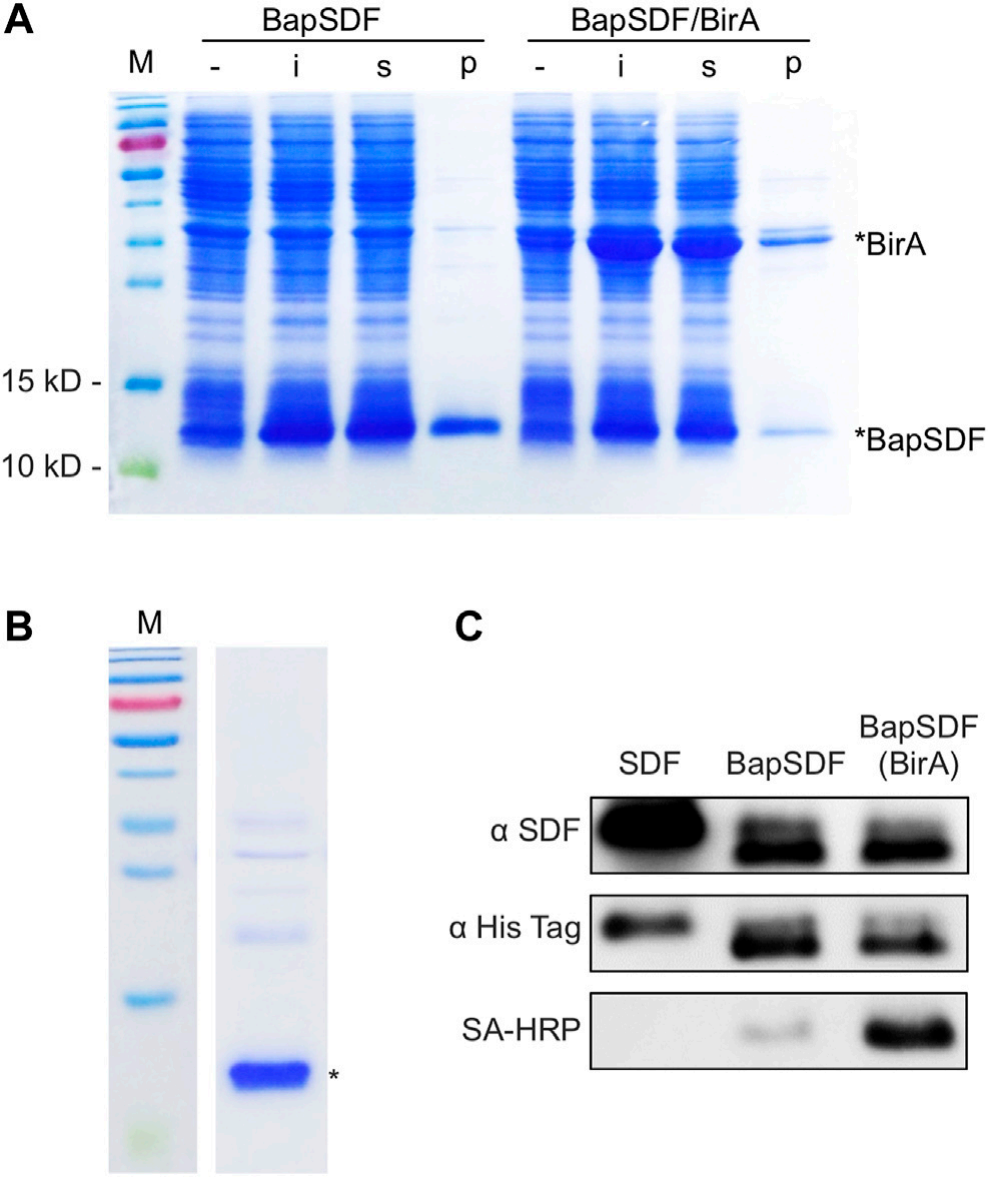
Expression and purification of BapSDF. **(A)** BapSDF and BapSDF co-expressed with BirA strains were induced by IPTG. Then, the cells were lysed, and different components were collected for detection. -, whole-cell extraction before induction; i, whole-cell extraction after IPTG induction; s, supernatant of the *i*th sample; p, precipitate of the *i*th sample. **(B)** Purified BapSDF co-expressed with BirA. **(C)** Western blotting was used to detect the levels of SDF, BapSDF, and BapSDF co-expressed with BirA using the SDF antibody, His-tag antibody, and HRP-conjugated Sa. BapSDF was purified, and biotinylated BapSDF was verified using HRP-conjugated Sa.

The expression of the BapSDF protein alone or with BirA was successfully induced in the *E. coli* expression strain, as shown in Figure 1. For the BapSDF-expressing strain, the target protein expression was induced, and it accounted for the largest proportion of the whole-cell protein, as more than half of the target protein was soluble (Figure 1A). For the BapSDF/BirA strain, the expression of these two proteins was induced simultaneously (Figure 1A). Moreover, the BirA protein was present in the largest proportion followed by BapSDF in this strain. The two proteins were found to be mostly soluble. The soluble proportion of BapSDF appeared to increase when BirA expression was co-induced when comparing the two strains with the same culture and induction conditions.

To optimal inducing condition, the effect of different concentrations and time of adding the inducer IPTG on BapSDF/BirA expression was determined. The results showed that a high concentration of IPTG (1 mM) impedes the expression of target proteins as compared to that with a low concentration (0.5 mM) under the same culture conditions (Supplementary Figure S1A). The strains were inoculated simultaneously in four flasks and cultivated for different time periods to reach a different amount of biomass before adding IPTG. The cells were induced for the same time, and the production of the target protein was estimated. Judging from the results of the SDS-PAGE analysis, young cells (with a low OD_600_ of 0.6) seemed to have the highest production of the target protein (Supplementary Figure S1B). SDF-1(alpha) is predicted to be a basic protein with an isoelectric point of approximately 10, and we investigated the dissolvability of BapSDF/BirA in various pH buffers. As shown in Supplementary Figure S1C, BapSDF had the largest soluble proportion in pH 11 buffer than other pH conditions (pH 7–10). We applied this pH buffer in the following purification step unless stated otherwise.

### Purification of BapSDF

Two BapSDFs were purified from the supernatant of the whole-cell lysate using nickel affinity chromatography. The BapSDF from the BapSDF/BirA strain was purified, resulting in low purity (Supplementary Figure S2). BirA seemed to be enriched along with BapSDF, although no His-tag was designed or predicted. BirA accompanied the main component of purified BapSDF. It is possible that BirA interacts with BapSDF and was co-purified with BapSDF by its affinity to nickel sepharose.

Denaturing agent urea was then used to break protein interactions, and finally, highly pure BapSDF was obtained (Figure 1B). The eluted target proteins were dialyzed, and BapSDF biotinylation by BirA was detected (Figure 1C). Two purified BapSDFs from individual and co-expression strains were successfully detected using the anti-His and anti-SDF-1(alpha) antibody. To test whether BapSDF was biotinylated by the co-expression of BirA, purified BapSDF from the co-expression strain was detected using HRP-conjugated Sa, suggesting that this protein possessed biotin (Figure 1C). Interestingly, BapSDF is not supposed to have SA-HRP binding ability because the expression plasmid of this *E. coli* strain does not contain the birA gene. It is supposed that the endogenesis BirA in host *E. coli* was expressed in low abundance and can conjunct biotin to partial Bap peptide residue. Therefore, the biotinylation of BapSDF by co-expression with BirA was successful, and biotinSDF was finally obtained.

### Assembly of SDF-MNP

**FIGURE 2:**
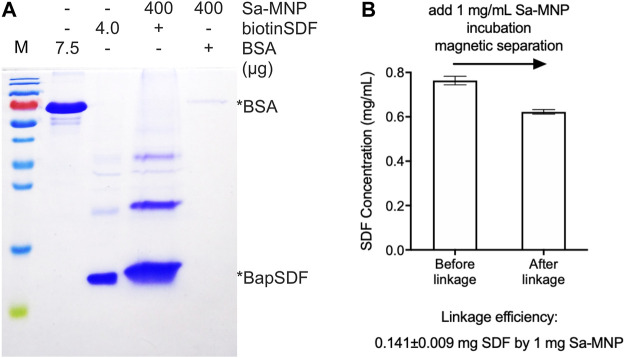
Assembly of SDF-MNP. **(A)** Estimation of loaded protein using Sa-MNP. BSA and purified BapSDF co-expressed with BirA were adjusted to 0.5 mg/ml, and then, Sa-MNP were added at a final concentration of 1 mg/ml. After sonication and incubation, assembled MNP were separated using magnetic separation and washed with protein storage buffer. Subsequently, 400 μg of assembled MNP were detected. **(B)** ELISA estimation of SDF concentration before and after capture by Sa-MNP. The maximum concentration of purified BapSDF (BirA) collected and used in the incubation mixture. Sa-MNP specifically captured BapSDF (BirA) with an efficiency of approximately 14% in two tests.

Biotinylated proteins or peptides specifically bind to the Sa-modified material because of the biotin–Sa affinity. BiotinSDF and Sa-MNP were incubated together, collected using magnetic separation, and detected using SDS-PAGE. BiotinSDF can be co-precipitated by Sa-MNP, whereas BSA was not detected (Figure 2A). These results suggest specific binding between biotinSDF and Sa-MNP.

The efficiency of BapSDF binding on the Sa-MNP surface was initially estimated by the reduction of BapSDF in the supernatant of the incubation system using ELISA. However, the results were found to exhibit a high variation in subsequent repeats. Thus, it was speculated that although most MNP were eliminated from the reaction system, the remaining MNP loaded with SDF-1(alpha) had a considerable effect on the measurements using ELISA. We then directly detected loaded SDF-1(alpha) on MNP using ELISA. We used 5% BSA to block the non-specific affinity of MNP during ELISA (Supplementary Figure S3B).The results were reproduced with a loading efficiency of approximately 14%, indicating that 1 mg of MNP can capture 0.14 mg of SDF-1(alpha) (Figure 2B, Supplementary Figure S3A). Since biotin–Sa is characterized by a strong affinity, it is speculated that the linkage of SDF with MNP by this method was irreversible.

### Physicochemical Properties of SDF-MNP

**FIGURE 3:**
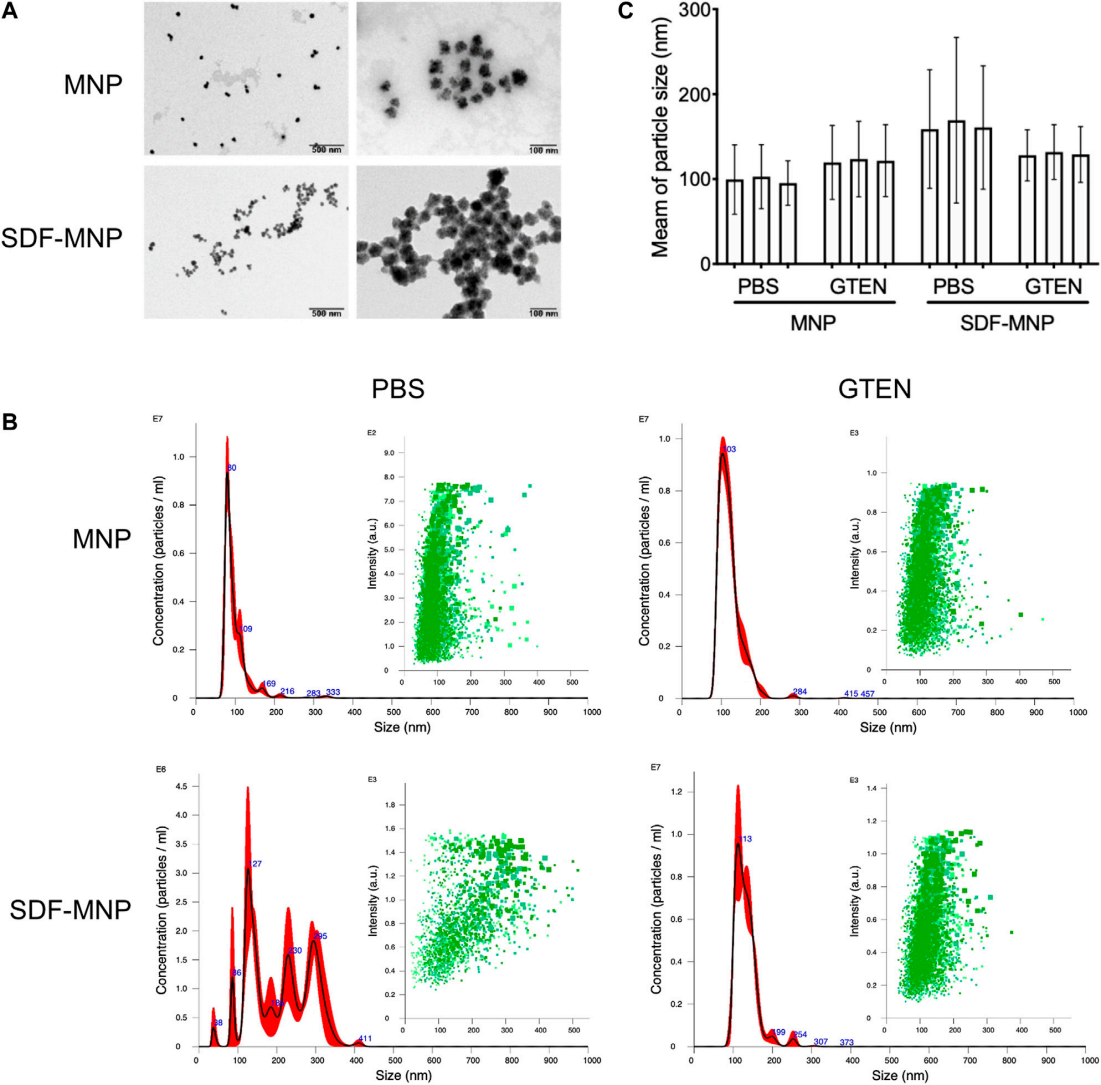
Characteristic of SDF-MNP. **(A)** TEM images of MNP and SDF-MNP. Scale bars: left, 500 nm; right, 100 nm. **(B)** Hydrate particle size–concentration histogram (red) and scatter plot (green) of MNP and SDF-MNP in PBS or GTEN buffer. **(C)** Statistical mean of particle size in every test. Particle size was measured in three tests.

To understand the characteristics of SDF-MNP, their preliminary physicochemical properties were investigated. The original MNP we bought had a particle size of 30 nm. MNP and SDF-MNP (originally suspended in PBS) were observed using TEM. MNP demonstrated good dispersibility, whereas SDF-MNP got aggregated. The distance between individual particles of SDF-MNP appeared closer than that between MNP (Figure 3A). The hydrate particle sizes were further estimated. The MNP/PBS suspension showed one main peak at 80 nm and mostly ranged from 50 to 150 nm. The size of MNP was 99.5 ± 36.4 nm, whereas that of SDF-MNP was 208.3 ± 79.2 nm. The SDF-MNP had multiple peaks ranging from 50 to 350 nm (Figures 3B,C; Supplementary Table S2). This was probably because of a mixture of MNP and SDF-MNP and/or their aggregation in this suspension. These results indicate that the characteristics of MNP are somewhat altered after the capture of SDF under the tested conditions. The aggregation of SDF-MNP may decrease their biofunction.

Since SDF was found to be stable in 5% GTEN storage buffer, we resuspended SDF-MNP in this buffer after their capture. The hydrate particle size of MNP-SDF/GTEN buffer showed one main peak with a maximum value at 113 nm, similar to the MNP/GTEN buffer with a maximum peak at 103 nm. The calculated size of MNP was 121.3 ± 30.4 nm and of SDF-MNP was 131.5 ± 28.2 nm (Figures 3B,C; Supplementary Table S2). These results indicate that SDF capture enlarges the hydrate particle size of MNP in either PBS or GTEN buffer, based on the size histogram or their mean sizes. The captured SDF was also found to contribute to the aggregation of MNP, which was eliminated by suspension in GTEN storage buffer, which may probably be explained by its different charge character in these two buffers.

### Promotion of Attachment and/or Uptake of SDF-MNP

**FIGURE 4:**
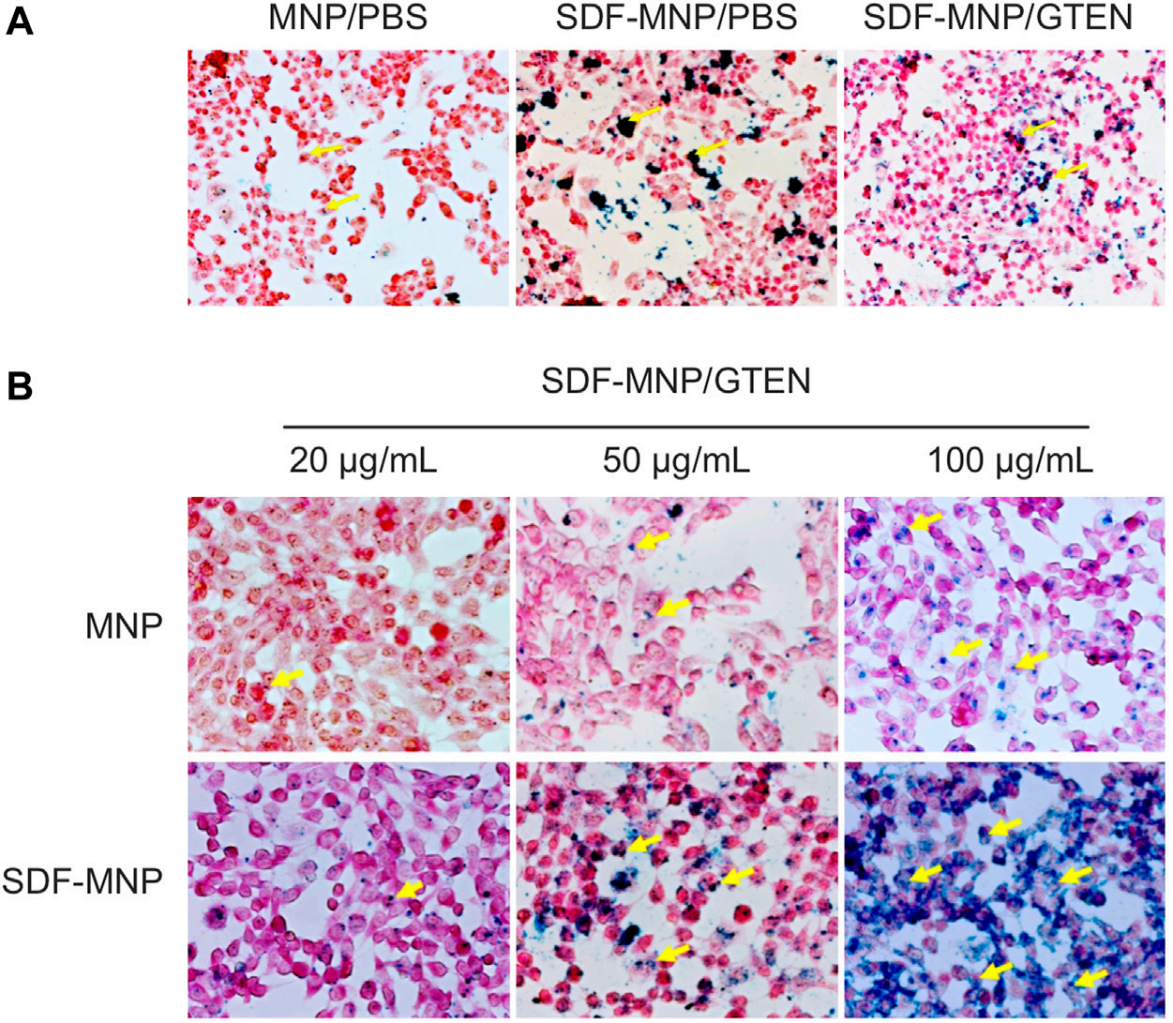
Perls stain of total attached iron or iron uptake of MSCs incubated with MNP or SDF-MNP (magnification: ×400). **(A)** SDF-MNP resuspended with PBS or GTEN and incubated with iPSC-MSCs. **(B)** iPSC-MSCs were treated with SDF-MNP/GTEN at different final concentrations. Then, total attached iron or iron uptake was determined using Perls reagent. Red signal is a stained cell, and yellow arrows pointing to dark blue signals represent iron. They showed a more obvious, well-distributed iron signal, which increased in a concentration dependent manner.

SDF binds primarily to CXCR4 on the membrane of iPSC-MSCs to induce intracellular signaling through several pathways and subsequent bioprocesses, including chemotaxis and cell proliferation (Girdlestone, 2016). Nanoparticles can be taken up by cells through endocytosis. To estimate the attachment/absorption of SDF-MNP, CXCR4-positive MSCs were co-cultured with SDF-MNP and observed under a microscope after Perls staining. The results showed that a few light blue signals reflecting the iron stack were captured when treated with MNP alone. Large dark crumb signals were observed after treatment with SDF-MNP suspended in either PBS or GTEN buffer, and the former demonstrated a strong aggregation of MNP (Figure 4A). This low dispersion in the SDF-MNP/PBS experiment was in accordance with the results mentioned before. We used SDF-MNP/GTEN for co-culture with iPSC-MSCs at different final concentrations. Ferric ion signaling on/in cells was found to increase gradually with an increase in concentration (final concentrations of 25, 50, and 100 µg/ml of MNP or SDF-MNP; Figure 4B). A remarkable increase in the iron stack of cells was seen after treatment with SDF-MNP as compared to that with MNP. The results implied that immobilized SDF contributed to the attachment or endocytosis of MNP by binding to CXCR4 on the MSCs membrane.

### Cytotoxicity of SDF-MNP

**FIGURE 5:**
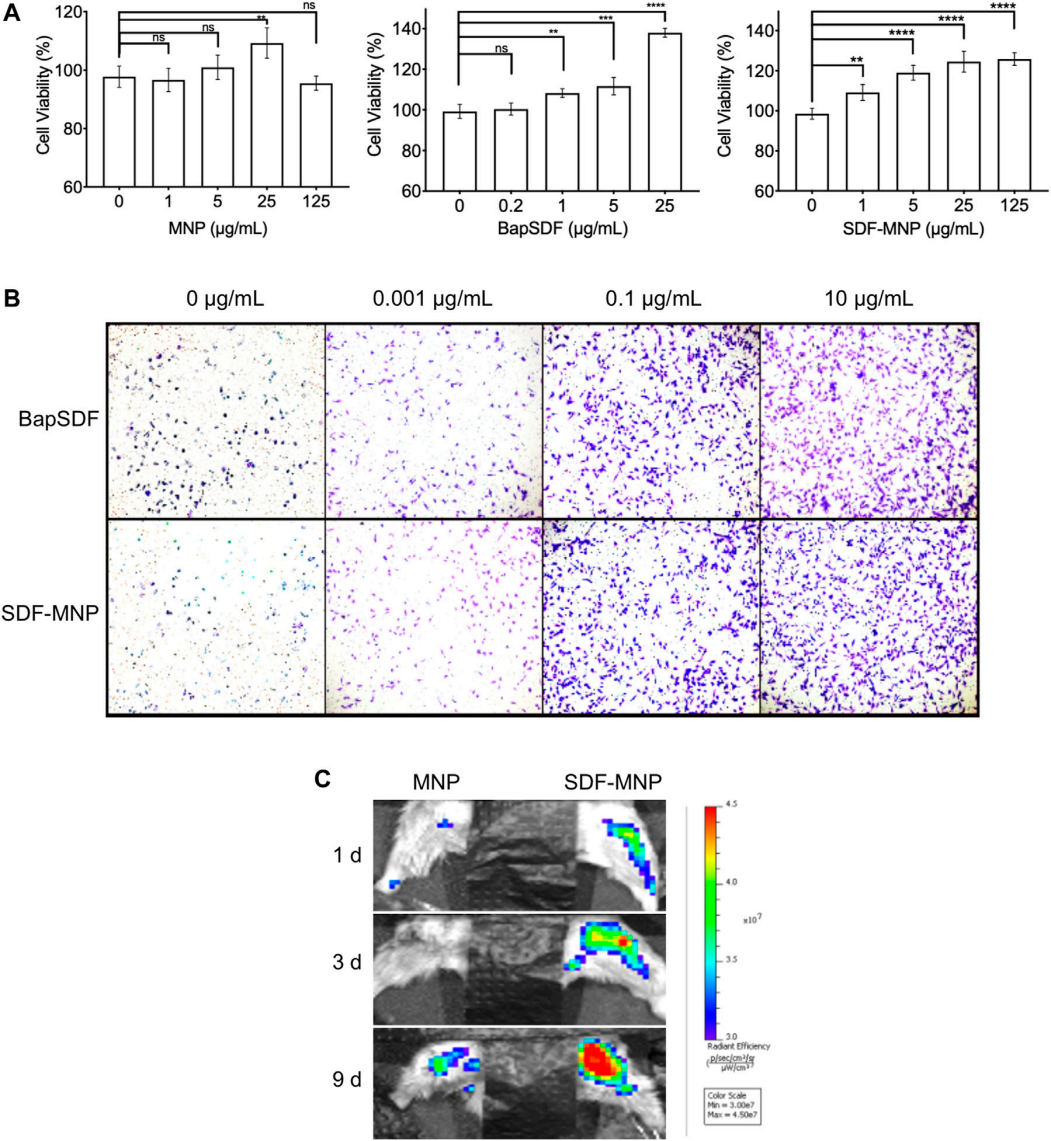
Toxicity and chemotaxis of SDF-MNP. **(A)** Viability of iPSC-MSCs treated with different concentrations of MNP, BapSDF, and SDF-MNP. **(B)** Transwell assay for iPSC-MSCs incubated with SDF-MNP. Cells that crossed the membrane and attached on the SDF-MNP side were stained using the crystal violent reagent (magnification: ×100). **(C)** Recruiting ability was estimated by the intensity of the labeled iPSC-MSCs at the bone fracture site. The number of aggregated iPSC-MSCs in SDF-MNP treatment (Right) increased from the blue to red signal while lower signal increasing for MNP without SDF treatment (Left). SDF-MNP showed low cell toxicity and demonstrated chemotactic ability for iPSC-MSCs.

The metabolic product of MNP may affect cellular homeostasis and proliferation. The cytotoxicity of free and immobilized SDF was determined using the CCK-8 assay and represented by cell viability. We further evaluated the effect of SDF-MNP on cell viability along with MNP and/or SDF (Figure 5A). The results showed that MNP had minimal effects on cell viability, even after treatment with 125 µg/ml MNP. SDF was found to promote cell viability up to approximately 1.4-fold at 25 µg/ml as compared to that in the control experiment. A similar promotion of cell viability was observed after treatment with SDF-MNP at 1–25 µg/ml. A high concentration of 125 µg/ml had a similar effect to that of 25 µg/ml, reaching over 1.2-fold, as compared to that with the control experiment (Figure 5A). These data suggested that SDF-MNP demonstrated barely any toxicity and slightly promoted cell proliferation, which may be due to the induction of the SDF-1(alpha)/CXCR4 axis.

### Biofunction of SDF-MNP in Recruiting iPSC-MSC

Functional SDF-1(alpha), as a chemotactic agent, attracts iPSC-MSC to the milieu with its high concentrations. To investigate its chemotaxis function, we performed a transwell experiment of iPSC-MSCs treated with SDF-MNP along with free SDF as a control. The results showed that iPSC-MSCs crossed the transwell chamber membrane after adding either SDF or SDF-MNP. The number of these cells increased with an increase in concentration. This effect was obvious at 0.1 µg/ml. The high concentration of 10 µg/ml showed a better chemotaxis effect in this test (Figure 5B). These results suggest a biofunction of both SDF and SDF-MNP in chemotaxis. This biofunction of SDF-MNP was also preliminarily explored *in vivo*. Labeled iPSC-MSCs were injected into mice via the tail vein. The femur was then fractured, and SDF-MNP were injected around this tissue followed by magnetic immobilization. Labeled iPSC-MSCs around the fractured tissue were then visualized and recorded (Figure 5C). The results showed that the signal around the fractured tissue injected with SDF-MNP gradually increased significantly compared to that after MNP administration, indicating that SDF-MNP immobilized in tissue can recruit more iPSC-MSCs. This result suggests an efficient chemotaxis by SDF-MNP on transplanted endogenous iPSC-MSCs *in vivo*.

In this study, we successfully constructed magnetic controlled, nano-scaled SDF-1(alpha) for stem cell-based repair. We linked SDF-1(alpha) to MNP using an Sa–biotin affinity system. We expressed and purified biotinSDF from a biological source, instead of chemically linking biotin with SDF-1(alpha). Then, the commercially obtained Sa-MNP were incubated with biotinSDF. After magnetic separation, SDF-MNP were collected. The loading efficiency reached approximately 14% under the tested conditions. This SDF-MNP showed better dispersibility in the GTEN buffer, mostly because of the charge characteristics. Attachment and/or uptake of SDF-MNP by iPSC-MSCs were found to be higher than those of MNP alone. This is probably due to the affinity between SDF-1(alpha) in SDF-MNP and CXCR4 expressed on the surface of iPSC-MSCs. Furthermore, the cell viability and transwell experiments suggested that SDF-MNP is a low-cytotoxic, bio-functional, nanochemokine that can recruit iPSC-MSCs.

## Discussion

The mobilization of a sufficient number of stem cells to the injured tissue site contributes to stem cell-based repair. SDF-1 is a cell chemokine that plays roles in the trafficking and homing of stem cells by recognizing the highly expressed marker CXCR4 (Lau and Wang, 2011). Interestingly, when pretreated with CXCL12 (SDF-1) the HSCs (hematopoietic stem cells) become stiffer and more rigid. This is suggested that, after being recruited by SDF-1, the stem cells bind with SDF-1 and are prone to retention (Du et al., 2019). Studies have shown that SDF-1-loaded scaffolds and other biomaterials have low toxicity and biocompatibility to recruit stem cells to target injury sites, along with promotion of VEGF and TGF-β secretion, which contributes to tissue regeneration (Zhang et al., 2010; Girdlestone, 2016).

Iron oxide nanoparticles (or magnetic nanoparticles) have long retention time in circulation, are mostly biodegradable, and have low cytotoxicity. They are bio-degradable in nature by various ways; they can get cleared by opsonins which can activate the complement system and then label particles to be taken up by phagocytic cells (Mehta, 2022). The most attractive characteristic of MNP comparing with microparticles such as liposomes, polymers, or carbon nanoshell is this nanodelivery material can carry cargos and allow them to be *in situ* by applying magnetic field (Ventola, 2012). Herein, we propose a simple strategy by immobilizing SDF-1 on magnetic nanoparticles and placing them on the target site to recruit more stem cells by enduring and high concentration SDF-1(alpha). The SDF-MNP around the injected site release to form gradient concentration to promote transplant and endogenesis of MSC homing by penetration from the vessel. SDF-1 specifically binding with CXCR4 on the surface of MSCs may have contribution in retaining MSCs by changing them into stiff and rigid status (Du et al., 2019). Even with good characteristics of MNP for loading and delivery, the immunobiological reaction by aggregation or disorder iron metabolism still need to be taken into consideration.

In this study, we purified biotin-modified SDF-1(alpha) by co-expression BirA. However, BirA can be co-purified with SDF-1(alpha) due to their interaction. To enhance the purity of the target protein, we utilized approaches while considering the following two concerns. First, for marked production after induction, BirA may bind non-specifically with nickel agarose. Second, BirA, as a biotin ligase, has a proximal interaction with the Bap peptide to exert biotinylation. Enhancing the binding volume of the supernatant implies that more BapSDF can occupy the nickel agarose gel and less BirA has non-specific binding during nickel chromatography. However, this approach did not contribute to an increase in BapSDF purity (Supplementary Figure S2). This implies that the two proteins were partially co-eluted owing to their proximal effects. We then utilized highly mild detergent concentrations (1% and 2% Triton-X 100) in the supernatant for incubation with nickel agarose, which still did not improve BapSDF purity. We finally successfully purified this SDF-1 exclusively under denature condition and refolded in (Kijowski et al., 2001) GTEN buffer condition.

Owing to the strict quality and endotoxic control, the price of commercially available SDF-1(alpha) is high (e.g., over 4,000 RMB per 10 μg from Merck); this cost is of SDF-1(alpha) without biotinylation. In addition, biotin was covalently bound to the Bap peptide amino acid residue in this study. By contrast, biotin modification by chemical methods is labeled as a functional group, which may affect protein activity. Subsequent purification of biotin-labeled proteins from the reaction system is costly and laborious. In our experience, approximately 8.5 mg biotin-labeled SDF-1(alpha) can be purified using nickel chromatography from 1 L of broth after adding 0.5 mM IPTG. This may reduce the cost and make the experimental procedure simple. In addition, we explored the storage of SDF-1(alpha), which preserves activated SDF-1(alpha) and SDF-MNP for further experiments.

Multiple cytokines contribute to stem cell-based therapy efficiency by promoting different stages of stem cell regeneration. It was found that SDF-1(alpha) restores angiogenesis synergistically with VEGF, which contributes to injury tissue repairing (Jin et al., 2013). Researchers have prepared an SDF-1(alpha) construct with a bone morphogenetic protein (BMP)-2 scaffold to test its role in bone regeneration. This construct promotes bone repair and regeneration (Wang et al., 2018). A similar study suggests that, in the future, co-assembled SDF-1(alpha) and BMP-2 using NapFFY (hydrogelator) hydrogel can replace bone transplantation for repairing of periodontal bone defects clinically (Yao et al., 2020). Since the SDF-MNP was demonstrated to be a promising material in our study, other functional factors with MNP can also be constructed. The combination of different factors by using a simultaneous or spatiotemporal release approach may further lead to the development of novel repair strategies.

## Data Availability

The original contributions presented in the study are included in the article/Supplementary Material; further inquiries can be directed to the corresponding authors.

## Abbreviations

BMP, bone morphogenetic protein; CXCR4, C-X-C motif chemokine receptor 4; MNP, magnetic nanoparticle; MSC, mesenchymal stem cell; SDF, stromal cell-derived factor; Sa, streptavidin; VEGF, vascular endothelial growth factor; TGF-β, transforming growth factor beta.
