# Supplementary material for: Magnetic Nano-Sized SDF-1 Particles Show Promise for Application in Stem Cell-Based Repair of Damaged Tissues
Source: Front Bioeng Biotechnol. 2022 Apr 27;10:831256. doi: 10.3389/fbioe.2022.831256 (PMC9091189; doi:10.3389/fbioe.2022.831256)
Supplement: Supplementary file 1 [file DataSheet2.DOCX]

# *Response Figure 1 (Response to reviewers).*


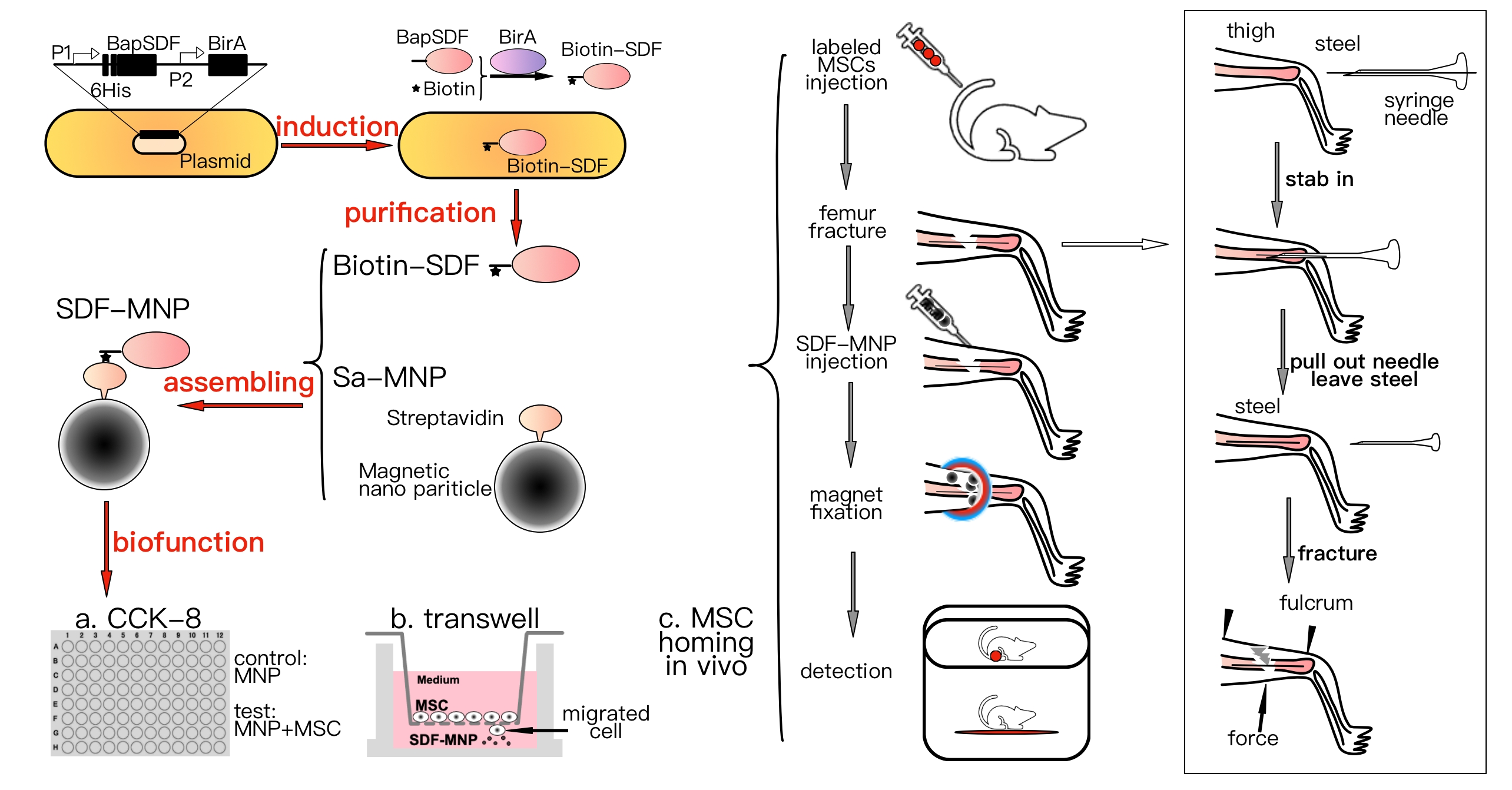


**Response Figure 1**. Schematic diagram of this study. Four main experiments including inducing biotin modified SDF-1, purification, assembling biotin-SDF and Sa-MNP, detection for its biofunction in vitro and in vivo were carried out (left, red font and arrow). Especial for femoral injured model in vivo test (right), the thin steel cross through syringe needle, then they were stabbed into femur marrow cavity. The needle was pulled out and left thin steel in. Gently fractured the femur in middle and the thin steel can keep two break bone together instead of total separated which is good for bone rebuilding. The labeled MSCs were transplanted by tail injection before femur fracturing. After injured model generated, the hollow ring magnetism was applied at the injection site for half an hour. The mice were imaged and signal was analyzed by using small animal in vivo imaging (SAIVI).
