# Supplementary material for: Magnetic Nano-Sized SDF-1 Particles Show Promise for Application in Stem Cell-Based Repair of Damaged Tissues
Source: Front Bioeng Biotechnol. 2022 Apr 27;10:831256. doi: 10.3389/fbioe.2022.831256 (PMC9091189; doi:10.3389/fbioe.2022.831256)
Supplement: Supplementary file 2 [file DataSheet1.DOCX]

Supplementary Material

# Supplementary Figures

#
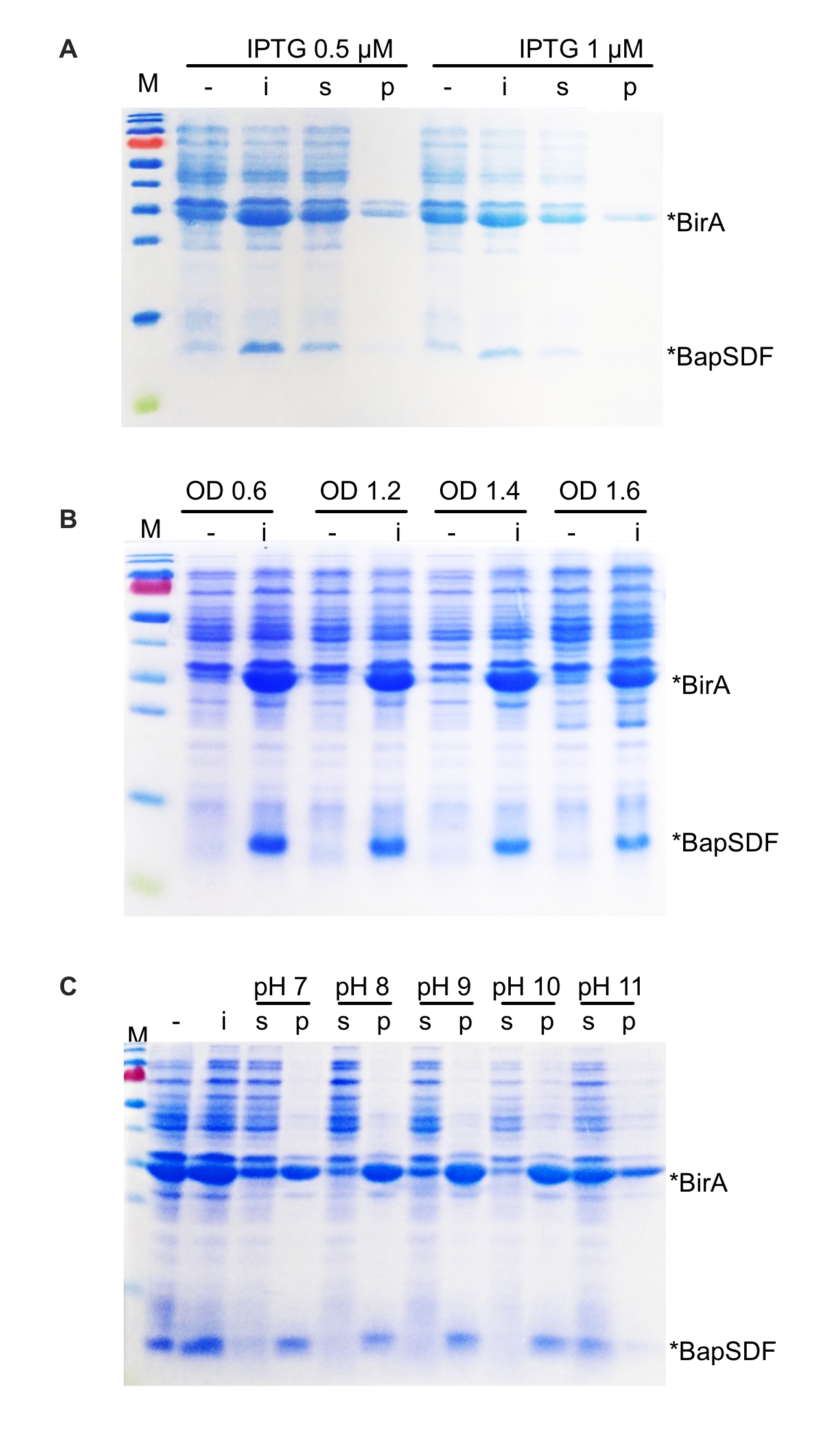


**Supplementary Figure 1**. Detection of BapSDF after the induction of BapSDF (BirA) in *Escherichia coli* BL21 . A: Production of BapSDF after induction by 0.5 and 1.0 mM IPTG. B: Final concentration of BapSDF after IPTG induction at growth media with different cell densities (measured as OD600 values). C: Soluble component detection in different pH lysis buffer solutions of the induced cells. BapSDF showed good solubility status after lysis in a pH 11 buffer. -, whole cell extraction before induction; i, whole cell extraction after IPTG induction; s, supernatant of i sample; p, precipitate of i sample. The addition of 0.5 mM IPTG after the medium reached an OD600 of 0.6 led to a higher production of BapSDF.


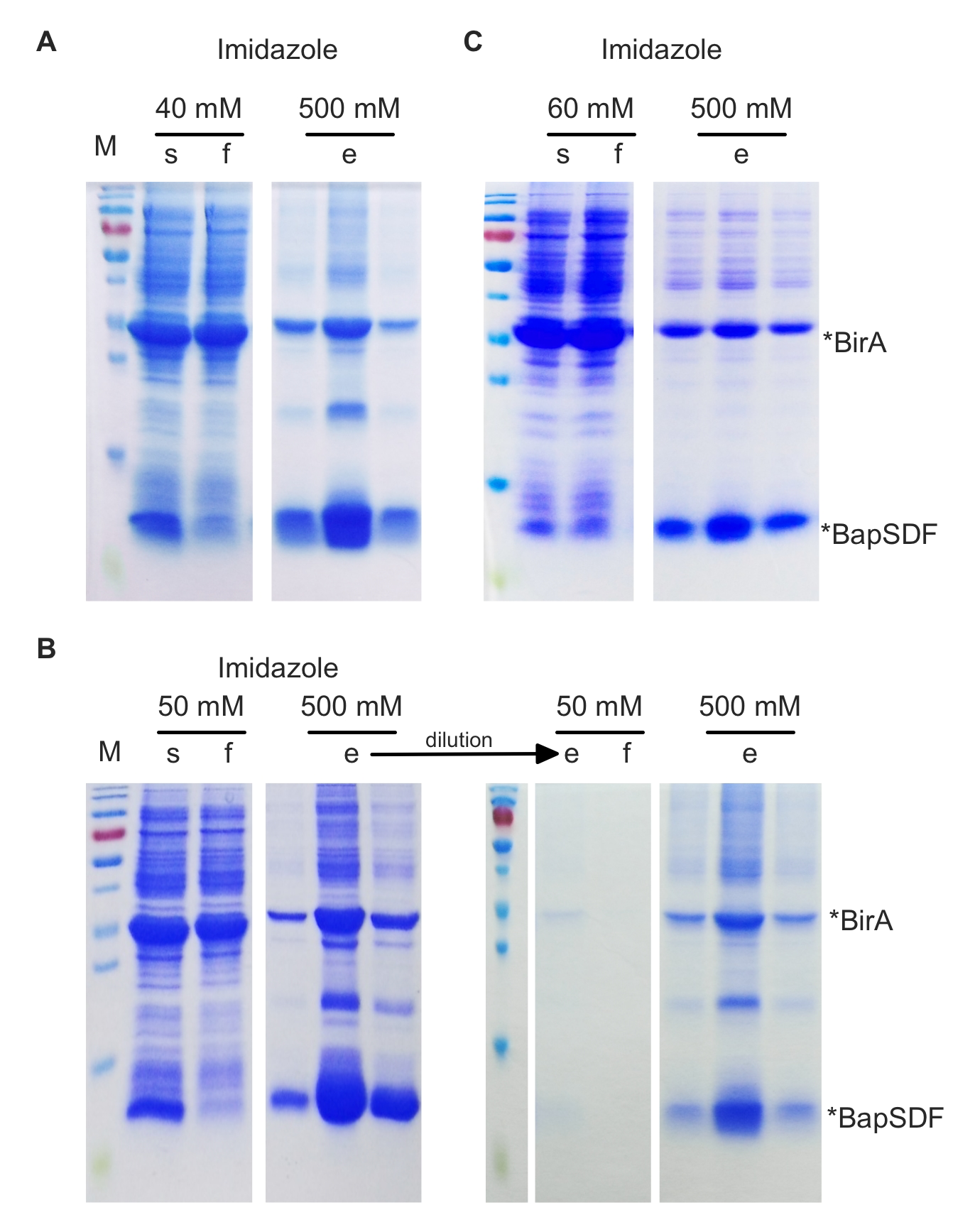


**Supplementary Figure** **2**. Purification of BapSDF by Nickel affinity chromatography using non-denaturing buffer. Following IPTG induction of the cells, the supernatant obtained following whole cell extraction was collected and subjected to Nickel chromatography. Then, after washing the gel with a low concentration of imidazole solution (A: 40 mM; B:50 mM; C: 60 mM), 500 mM imidazole buffer was added for the elution of the bound protein. This step was repeated three times. s: supernatant of the whole cell extract; f: flow of supernatant through Nickel chromatography; e: elution by 500 mM imidazole buffer. In Figure B, the eluent was diluted with 50 mM imidazole and bind with Nickel chromatography and then eluted. All samples were detected by SDS-PAGE. Under all testing conditions, BirA was found to be co-purified with BapSDF.


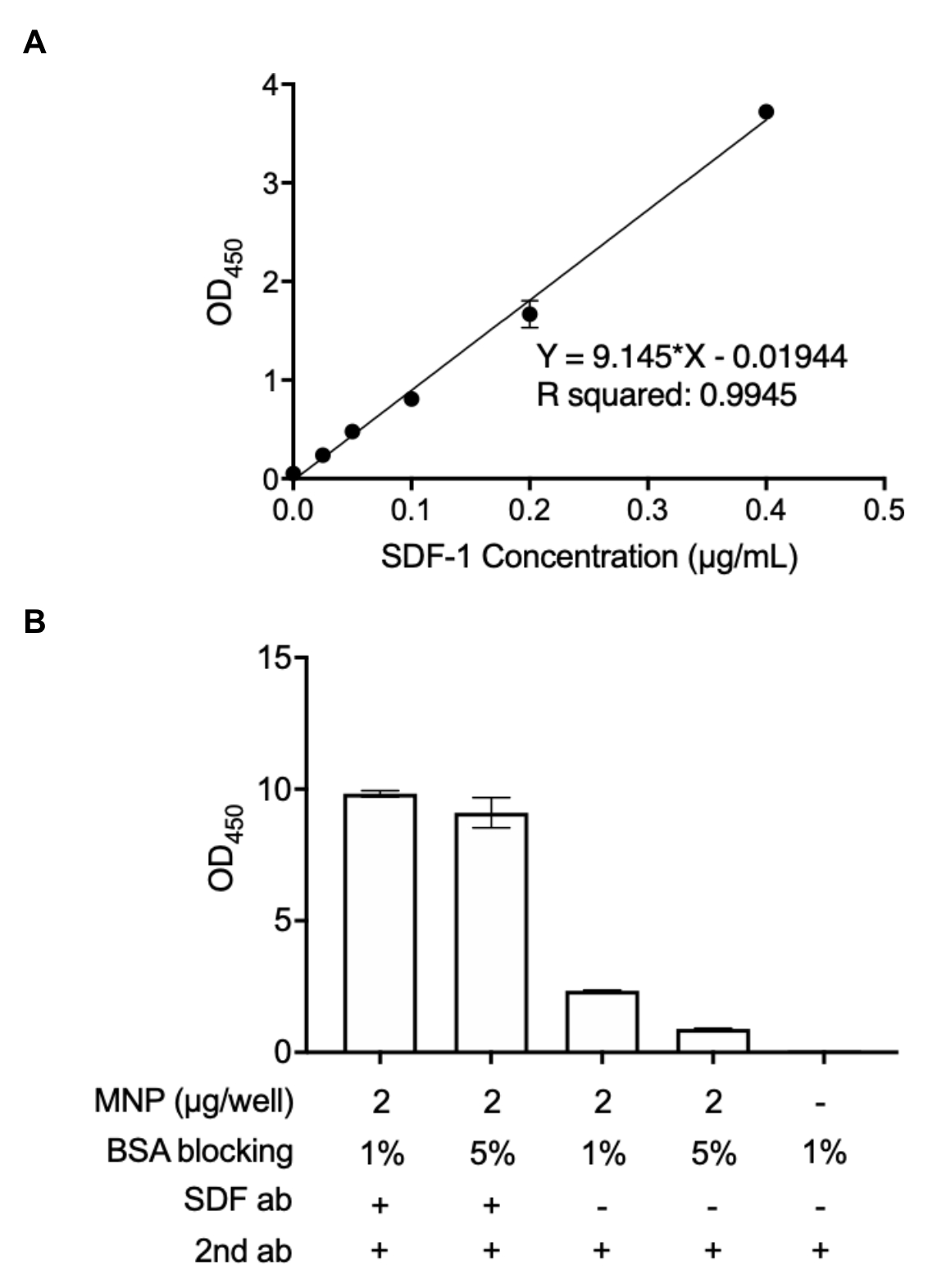


**Supplementary Figure** **3**. ELISA of BapSDF and MNP. A: standard curve of BapSDF concentration (OD measured at 450 nm). R squared: 0.9945. The standard curve showed credible linear regression. B: OD450 values of different MNP-treated media. This test suggested MNP to have a non-specific affinity and 5% BSA blocking could reduce but not eliminate the non-specific binding.

# Supplementary Tables

**Supplementary** **Table 1**. Primers and plasmids used in this study.

| Primers | Sequence | Discription |
| --- | --- | --- |
| bapsdf-1 | F: cgGAATTCgGGCCTGAACGATATTTTTGAAGCGCAGAAAATTGAATGGCATATGAAACCAGTCAGCCTGAGC | Red: EcoR I enzyme digest DNA; green for bap DNA |
|  | R: ataagaatGCGGCCGCTTACTTGTTTAAAGCTTTCTCCAGGTACTC | Red for Not I enzyme digest DNA |
| birA | F: gaAGATCTcATGAAGGATAACACCGTGCCA | Red for Bgl ll enzyme diagest DNA |
|  | R: ccgCTCGAGTTATTTTTCTGCACTACGCAGGG | Red for Xho I enzyme digest DNA |
| sdf-1 | F: ccgCTCGAGcaAAACCAGTCAGCCTGAGCTACC | Red for Xho I enzyme digest DNA |
|  | R: ccgCTCGAGTCACTTGTTTAAAGCTTTCTCCAGG | Red for Xho I enzyme digest DNA |

| Plasmid | Discription |
| --- | --- |
| pUC19-T-sdf-1 | plasmid containing sdf-1 dna |
| pUC19-T-bapsdf-1 | plasmid containing bapsdf-1 dna |
| pUC19-T-birA | plasmid containing birA dna |
| pET28a(+)-sdf-1 | expression plasmid containing sdf-1 |
| pCDFDuet-1-bapsdf-1 | expression plasmid containing bapsdf-1 dna in MCS (multiple clone site) 1 |
| pCDFDuet-1-bapsdf-1-birA | expression plasmid containing bapsdf-1 dna in MCS1, birA dna in MCS2 |

**Supplementary Table 2**. Summary of the concentration and size of particles.

| Samples | | MNP | | SDF-MNP | |
| --- | --- | --- | --- | --- | --- |
|  |  | PBS | TENG | PBS | TENG |
| Test 1 | Concentration particles (/mL) | 3.21E+08 | 1.72E+09 | 2.48E+08 | 1.46E+09 |
|  | Mean (nm) | 99.5 | 119.6 | 158.9 | 128 |
|  | SD | 40.9 | 43.4 | 69.9 | 30.2 |
|  | Mode (nm) | 89.1 | 95.3 | 94.9 | 112.2 |
| Test 2 | Concentration particles (/mL) | 2.96E+08 | 1.56E+09 | 2.30E+08 | 1.75E+09 |
|  | Mean (nm) | 102.8 | 123.6 | 169.4 | 131.8 |
|  | SD | 37.8 | 44.6 | 97.5 | 32.3 |
|  | Mode (nm) | 79.7 | 96.8 | 84.7 | 112.1 |
| Test 3 | Concentration particles (/mL) | 2.37E+08 | 1.65E+09 | 3.55E+08 | 1.94E+09 |
|  | Mean (nm) | 95.3 | 121.7 | 160.8 | 129 |
|  | SD | 26.2 | 42.3 | 72.6 | 32.8 |
|  | Mode (nm) | 79.6 | 97 | 121.3 | 103.9 |
